# Pathophysiological Effects of Synthetic Derivatives of Polymeric Alkylpyridinium Salts from the Marine Sponge, *Reniera sarai*

**DOI:** 10.3390/md12052408

**Published:** 2014-04-30

**Authors:** Marjana Grandič, Robert Frangež

**Affiliations:** 1 Institute for Hygiene and Pathology of Animal Nutrition, Veterinary Faculty, University of Ljubljana, Cesta v Mestni log 47, Ljubljana 1000, Slovenia; E-Mail: marjana.grandic@vf.uni-lj.si; 2 Institute of Physiology, Pharmacology and Toxicology, Veterinary Faculty, University of Ljubljana, Gerbičeva 60, Ljubljana 1000, Slovenia

**Keywords:** alkylpyridinium compounds, APS12-2, APS3, cardiotoxicity, hemolysis, nicotinic acetylcholine receptors, neuromuscular junction, mouse, rat, synthesis

## Abstract

Polymeric 3-alkylpyridinium salts (poly-APS) are among the most studied natural bioactive compounds extracted from the marine sponge, *Reniera sarai*. They exhibit a wide range of biological activities, and the most prominent among them are the anti-acetylcholinesterase and membrane-damaging activity. Due to their membrane activity, sAPS can induce the lysis of various cells and cell lines and inhibit the growth of bacteria and fungi. Because of their bioactivity, poly-APS are possible candidates for use in the fields of medicine, pharmacy and industry. Due to the small amounts of naturally occurring poly-APS, methods for the synthesis of analogues have been developed. They differ in chemical properties, such as the degree of polymerization, the length of the alkyl chains (from three to 12 carbon atoms) and in the counter ions present in their structures. Such structurally defined analogues with different chemical properties and degrees of polymerization possess different levels of biological activity. We review the current knowledge of the biological activity and toxicity of synthetic poly-APS analogues, with particular emphasis on the mechanisms of their physiological and pharmacological effects and, in particular, the mechanisms of toxicity of two analogues, APS12-2 and APS3, *in vivo* and *in vitro*.

## 1. Introduction

Polymeric 3-alkylpyridinium salts (poly-APS) are one of more than 80 biologically active compounds found in several marine sponges of the order, Haplosclerida [1,2,3,4]. They have been isolated from crude extracts of the Mediterranean marine sponge, *Reniera sarai*. Poly-APS have been reported to comprise two polymers with molecular weights of 5520 and 18,900 Da, corresponding to 29 and 99–100 covalently, head-to-tail linked *N*-butyl-3-butyl pyridinium monomers [5]. In water solutions, they form larger supramolecular aggregates [5,6,7]. However, recent analyses have indicated that poly-APS are composed of one monomeric species only, with a molecular weight of 5520 Da [8].

Poly-APS are water-soluble compounds with high degrees of association and a broad spectrum of interesting biological activities [4,6]. These include hemolytic, cytolytic and cytotoxic activities [6], antifouling [9,10] and antimicrobial properties, including antibacterial [11] and anti-algal activities [12]. Poly-APS are also very potent, irreversible acetylcholinesterase (AChE) inhibitors [13,14,15]. Due to their ability to induce transient pore formation in biological membranes [16,17], poly-APS have been used for stable transfection of various mammalian cells with heterologous DNA and, thus, have a potential in gene therapy [18,19,20]. Moreover, poly-APS exert selective cytotoxicity against non-small cell lung cancer (NSCLC) cells, which are the most common form of lung cancer, and express α7-nicotinic receptors [21,22,23]. Cytotoxic concentrations of poly-APS are in the nanomolar range (0.36–0.86 nM) [23] and are much lower than the calculated concentrations in blood plasma inducing toxic and lethal effects after intravenous (i.v.) compound application. Toxic effects on mammals, arising from poly-APS interference with the cholinergic system, have been observed following administration of low doses (0.7 mg/kg) of poly-APS. At higher doses, these effects were masked by the more pronounced lethal activity of the compound related to hemolysis and platelet aggregation. The half-lethal dose (LD_50_) of poly-APS in rats has been estimated to be 2.7 mg/kg ([24], reviewed in [25]). Poly-APS have recently been shown, at a 1 μM concentration, to diminish endothelium-dependent relaxation of isolated rat thoracic aorta and to significantly decrease coronary flow in the heart [26].

Such biological effects of natural poly-APS and their possible application in the fields of industry (as components of environmentally friendly antifouling paints) and medicine (as new anti-cholinergic, transfection and chemotherapeutic agents) have led to the synthesis of several 3-alkylpyridinium analogues (sAPS) with different degrees of polymerization and different lengths of the constituent alkyl chains [27,28,29]. The synthesis of structurally well-defined analogues with different chemical properties and degrees of polymerization has enabled the regulation of the biological activities of sAPS.

The aim of this review is to summarize current knowledge on the biological activities and toxicity of sAPS, with particular emphasis on mechanisms of toxicity of two synthetic analogues, APS12-2 and APS3, *in vivo* and *in vitro*.

## 2. Synthetic Analogues of Polymeric Alkylpyridinium Salts

Their interesting biological effects, their potential use in the pharmaceutical and chemical industries, coupled with the insufficient quantities of natural poly-APS, have contributed to the development of new methods for synthesizing poly-APS analogues. This could enable the commercial production of sAPS with modified characteristics. In 2004, Mancini and colleagues synthesized dimers and tetramers of 3-alkylpyridinium salts [27]. In 2010, Houssen and colleagues reported a new protocol enabling synthesis of larger polymers that possess greater biological activities [28]. To determine how the structure of sAPS influences the biological activities, several sAPS, with various lengths of the alkyl chain, numbers of pyridinium rings and with different counter ions (bromide or chloride), have been synthesized.

**Figure 1:**
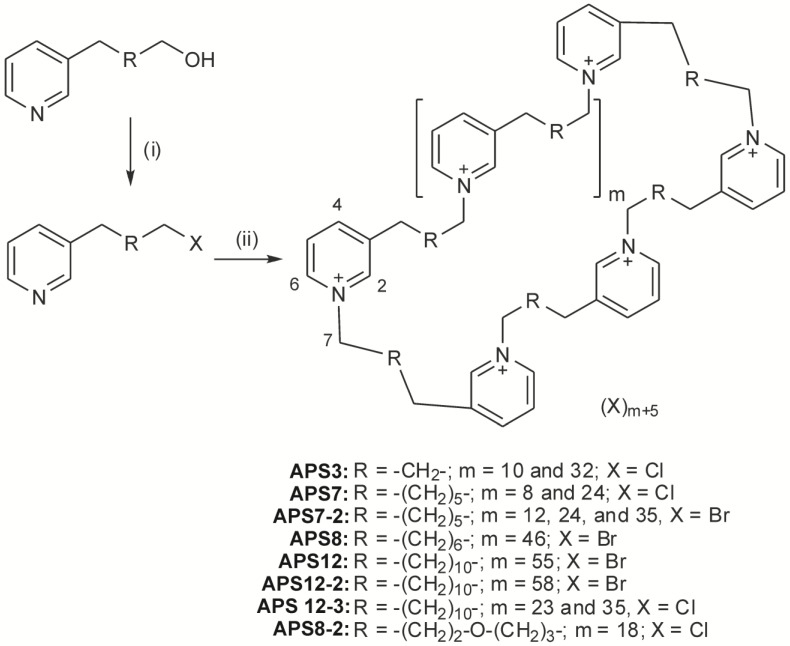
Synthesis of poly-(1,3-alkylpyridinium) salts. Reagents and conditions: for R = alkyl chain: (i) HBr, toluene, reflux overnight followed by neutralization to yield products with X = Br; thionyl chloride, dichloromethane, room temperature to yield products with X = Cl; (ii) reflux in acetonitrile or methanol (in the presence of a small amount of KCl for monomeric chloride), followed by microwave irradiation at 130 °C for the time length stated for each compound under the experimental section. Adapted from Zovko *et al.* [29], with permission from © 2012 Elsevier Ltd.

### sAPS Synthesis

A method that enables simple, rapid and affordable synthesis of highly purified alkylpyridinium compounds with a high degree of polymerization was developed [28,29]. Monomers were prepared according to a small modification of the method described by Davies-Coleman in 1993 [30]. Pyridyl alcohol was produced by coupling bromo-alcohol with 3-picoline. Bromide monomers were produced by neutralization of the alcohol treated with hydrogen bromide, while chloride monomers were produced by reacting the substrate with thionyl chloride. The monomers were further oligomerized in the presence of acetonitrile and methanol. Polymers were then formed using microwave-assisted polymerization. Their length depended on the time of irradiation [28,29]. Interestingly, the critical micelle concentration of selected sAPS (APS7, APS8 and APS12-2) was found to be above 1 mg/mL [31], e.g., considerably higher than that determined for natural poly-APS [5].

The chemical synthesis of poly-(1,3-alkylpyridinium) salts is shown in Figure 1.

The method is quick, safe, economical, eco-friendly and enables the production of large amounts of product [32]. Several sAPS have been produced with various degrees of polymerization, different cations and different lengths of the alkyl chain. Some analogues are mixtures of polymers with different degrees of polymerization. The basic chemical properties of the most studied sAPS are presented in Table 1.

**Table 1:** Basic chemical properties of polymeric 3-alkylpyridinium salts (poly-APS) and their synthetic analogues.

Compound	No. of Alky lC-atoms	No. of Polymers and Molar Ratio	Molecular Weight (kDa)	Degree of Polymerization	Counter Ion	Reference
Poly-APS	8	1	5.52	29	Cl^−^	[6]
APS3	3	2 (9:1)	1.46 (1.2/3.8)	10 and 32	Cl^−^	[29]
APS7	7	2 (2:1)	2.33 (1.4/4.2)	8 and 24	Cl^−^	[29]
APS8	8	1	11.9	63	Br^−^	[28]
APS12	12	1	12.5	51	Br^−^	[28]
APS12-2	12	1	14.7	60	Br^−^	[28]

## 3. Biological Activities of sAPS

### 3.1. Hemolytic and Antimicrobial Activity

Like natural poly-APS, the synthetic analogues (sAPS) have structures similar to those of cationic detergents [33]. The hemolytic activity for both is directly proportional to the length of the alkyl chain and the degree of polymerization [34,35]. The hemolytic activity of analogues with low molecular weights is very low or negligible [28,29,31]. The nature of the counter ion does not influence the hemolytic activity [29]. The electrophysiological effects of mono-, di- and tetra-meric sAPS [27] were evaluated also on cultured hippocampal neurons [17]. Here, again, low-molecular sAPS were found to be much weaker pore formers than the natural poly-APS, indicating that the polymerization degree and the subsequent formation of the supermolecular structure are crucial for the observed membrane activity.

sAPS possess antimicrobial properties and have proven to be more effective against Gram-positive (*S. aureus*) than Gram-negative bacteria (*E. coli*). The latter are more resistant to sAPS action, probably due to the additional lipopolysaccharide layer on the cells [27,29]. Their antibacterial activity increases with the increasing number of positive charges and the length of the alkyl chain. sAPS with a bromide counter ion are more active than sAPS with a chloride counter ion [11,29]. Interestingly, all sAPS, except APS3, which is the smallest, have higher antibacterial activities than natural poly-APS [29]. Compared with structurally similar compounds, like cetylpyridinium chloride (CPC), which has minimal inhibitory concentrations (MIC) for *S. aureus* and *E. coli* of <1.47 μM and 470 μM, sAPS are quite effective, their antibacterial activity against *E. coli* being greater (MIC_APS-12-2_ = 34.01 μM) and against *S. aureus* being comparable (MIC_APS-12-2_ = 6.8 μM) to that of CPC [29].

sAPS also inhibit the growth of pathogenic fungi; the length of the alkyl chain and the degree of polymerization are important. APS12-2, the analogue with the longest alkyl chain and the highest degree of polymerization, has the highest antifungal activity [29]. The effectiveness of several sAPS has been compared with that of some standard antifungal drugs. The antifungal activity of analogues APS12-2 and APS3 was similar to that of miconazole, while other antifungal drugs were ten to a hundred times more effective than sAPS [29]. sAPS, especially those with longer alkyl chains, are also effective against saprophytic fungi. The oxygen atom in the alkyl chain of APS8 significantly decreases its effectiveness. However, APS12-3 is appropriate as a biocide for protecting wood against the fungus, *Gloeophyllum trabeum* [29]. Finally, sAPS oligomers and polymers have the ability to effectively inhibit the settling of the marine barnacle, *Amphibalanus amphitrite*, larvae and are thus interesting as antifouling agents [36,37].

### 3.2. Effects of sAPS on Acetylcholinesterase

The most prominent biological activity attributed to sAPS is probably the inhibition of AChE, the enzyme in the nervous system synapses that hydrolyses the neurotransmitter, acetylcholine (ACh). Hydrolysis of ACh takes place at the bottom of a 2 nm-deep enzyme active site gorge, where the anionic site responsible for choline recognition and the catalytic site with its active serine are located. At the rim of the gorge, there is another binding site for the substrate and other ligands, called the peripheral anionic site [38]. This is also the binding site for natural poly-APS. The first non-competitive binding of poly-APS is followed by several successive phases ending in the irreversible inhibition of the enzyme, which is due to the aggregation and precipitation of AChE [13,14].

Unlike the natural poly-APS, the time-course of AChE inhibition by sAPS12 and APS12-2 is linear, showing the reversibility of inhibition [28]. This compounds act as noncompetitive AChE inhibitors, by binding to the peripheral anionic site and preventing the binding of ACh inside the enzyme gorge. It is assumed that binding takes place at this site, because the size of the synthetic analogues is too great to allow entry to the enzyme gorge, as these sAPS are very potent AChE inhibitors, acting in picomolar concentrations [28]. They could be used in medicine as drugs for treating conditions in which ACh secretion is reduced, *i.e.*, Alzheimer’s disease, myasthenia gravis and eye glaucoma [39].

### 3.3. Antitumor Activity of sAPS

Recent studies with synthetic analogue APS8 have shown that it is a potent inhibitor of α7-nicotinic receptors, at concentrations of less than 1 nM [40]. Since this concentration is lower than the inhibition constant for AChE (1.88 nM), APS8 activity is probably due to the inhibition of receptors and not AChE. APS8 inhibits the growth of various cancer cell lines, like A549 and SKMES-1, but is not toxic for normal fibroblasts [40]. Moreover, using flow cytometry and differential staining, it was found that APS8 triggers the apoptosis of cancer cells in a concentration-dependent manner [40]. This effect may be due to the antagonistic effect of APS8 on α7-nicotinic receptors, which are particularly abundant in various tumor cells of the respiratory tract in contrast to non-cancer cells [40]. The apoptosis caused by APS8 involves both intrinsic and extrinsic pathways and is activated by cell stress. In the extrinsic pathway, the death receptors are involved and are activated after binding certain ligands. A number of reactions are triggered, ultimately leading to apoptosis [40]. The results suggest that APS8 or similar compounds could be considered as promising compounds for antitumor drugs development for some types of lung cancer [40].

The basic biological activities of the most studied sAPS are summarized in Table 2.

**Table 2:** Biological activities of poly-APS and their synthetic analogues.

Compound	AChE Inhibition—*K_i_* (nM) *	Hemolysis (s^−1^ at 500 nM) **	IC_50_ for NSCLC (μM) ***
Poly-APS	irreversible inhibition	0.05	4.41
APS3	85	0	3000
APS7	10	0.1	480
APS8	1.875	2.6	478
APS12-2	0.036	5.0	470

NSCLC, non-small cell lung cancer; * [28]; ** [31]; *** [41].

## 4. Toxicity of APS12-2 and APS3

In view of the possible use of sAPS in medicine and the pharmacy setting, it was essential to evaluate their effects on mammals and to explore the mechanisms of their toxicity. APS12-2 and APS3 are the most studied sAPS. They were chosen for research due to the different mechanisms of their toxicity and their different chemical properties, which could account for their physiological, toxicological and pharmacologic activities.

APS12-2 is an analogue with a higher degree of polymerization and a longer alkyl chain, bearing a bromide counter ion. It is strongly hemolytic and acts as a non-competitive AChE inhibitor. APS3 is smaller and shorter, with a chloride counter ion. It is non-hemolytic and acts as a competitive AChE inhibitor. *In vivo* and *in vitro* experiments have provided significant data on the possible adverse effects of APS12-2 and APS3 on the vital functions of mammalian organisms, related to their effects on organ systems, organs, tissues and cells, as well as on the molecular level, as described below.

### 4.1. In Vivo Effects of APS12-2 and APS3

Before performing *in vivo* experiments, the median lethal dose for both sAPS analogues was estimated in Balb/c mice. Different doses of APS12-2 and APS3 were administered intravenously to male Wistar rats. Blood pressure, respiratory activity and electrocardiograms (ECG) were monitored. At the end of each experiment, vital organs were removed for histological analysis. The estimated median lethal doses for APS12-2 and APS3 in mice were 11.5 and 7.25 mg/kg [8,42]. Compared to natural poly-APS, with an estimated LD_50_ in rats of 2.7 mg/kg [24], the toxicity of APS12-2 and APS3 is low. In *in vivo* experiments, it was found that rats are more sensitive to both analogues than mice [8,42].

Sublethal effects of APS12-2 *in vivo* were determined in rats (sublethal doses of four and 5.5 mg/kg) to provide more understanding of the mechanistic specificity of this APS. i.v. application leading to mild transient bradycardia similar to that described for poly-APS above, but in this case, the heart rate gradually recovered. Arterial blood pressure (aBP) decreased significantly immediately following application. This was followed by a transient increase, then finally, a gradual return to the basal value. The bradycardia produced by the anticholinergic activity of the compounds, the hyperkalemia or the lung reflexes may be responsible for the reduction in aBP. The subsequent increase in aBP could be the consequence of a compensatory increased sympathetic tone as a response to the hypotension or the direct or indirect effect of the substance on peripheral blood vessel resistance. The fact that no increase in heart beat frequency was observed during the period of transient hypertension supports this view. Sublethal doses of APS12-2 also caused significant elevation of blood potassium levels, which could be an important cause of the cardiorespiratory toxicity of APS12-2 [8].

In rats, the death caused by a lethal dose (11.5 mg/kg) of APS12-2 was due to cardiorespiratory arrest [8]. Since the latter can be produced at plasma potassium concentrations above 10 mM [43,44,45], the cardiotoxic effects of APS12-2 may be related to its hemolytic activity and hyperkalemia (10.44 ± 0.44 mM) [8,31]. Respiratory arrest could be produced by the stimulation of juxtapulmonary capillary receptors in lung parenchyma [46,47]. These receptors are mechano-sensitive and are therefore activated by conditions, like pulmonary edema, congestion or pulmonary microembolism [48]. This could be the mechanism of respiratory arrest produced by lethal doses of APS12-2. This explanation is supported by histopathological findings of acute lesions observed in the pulmonary vessels of rats, the lysis of aggregated erythrocytes within their lumina and pulmonary edema [8]. APS3 was not lethal in experimental rats at doses up to 20 mg/kg and at cumulative doses up to 60 mg/kg. Only transient changes in blood pressure were observed. The serum potassium level was, as expected, not significantly altered, due to the absence of APS3 hemolytic activity [42]. *In vivo* experiments with APS3 further confirmed the putative role of hyperkalemia in the cardiotoxic activity of APS12-2.

The effects of APS12-2 and APS3 on *in vivo* measured parameters are summarized in Table 3.

*In vivo* experiments with APS 12-2 on mice, injected (2.2 μg/kg) intramuscularly at the base of the tail, showed that it decreased the compound muscle action potential (CMAP) [49]. Similar time- and dose-dependent reversible effects on CMAP amplitude were observed in mice after administration of APS3 at sublethal doses (0.3–3 mg/kg). Administration by i.v. of cumulative doses of APS3 (up to 60 mg/kg) in rats produced dose-dependent inhibition of nerve-evoked muscle contraction with an ID_50_ of 37.25 mg/kg. Since APS3 is a water-soluble substance composed of two relatively small polymers in a molar ratio 9:1,with molecular weights (m.w.) of 1.2 and 3.8 kDa, in contrast to APS12-2 (m.w. 17.7 kDa), it can pass the slit-pore in muscle capillary membranes, reach the postsynaptic membrane of the neuromuscular junction and cause neuromuscular block. The relative permeability of skeletal muscle capillary pores to substances with molecular weights of 342 and 5000 Da is 0.4 and 0.2.

**Table 3:** The effects of APS12-2 and APS3 on significant parameters in rats.

Measured Parameters	APS12-2 *	APS3 **
LD_50_ (mice)	11.5 mg/kg	7.25 mg/kg
ECG (rats)	bradycardia second degree atrioventricular block Ventricular extrasystoles	Transient tachycardia
Arterial blood pressure	Steep decrease immediately after application	First a decrease, then an increase above base-line value
Breathing	Respiratory arrest soon after application	No effect
Biochemical parameters	Statistically significant increase in K^+^ level (10.44 ± 0.44 mM)	Statistically significant increase in K^+^ level (5.66 ± 0.37 mM)
Muscle contraction	No effect up to 8.6 mg/kg	ID_50_ = 37.25 mg/kg

LD50, half-lethal dose; ID_50_, median inhibitory dose; ECG, electrocardiography; * [8]; ** [42].

In contrast to APS3, APS12-2 (at 11.5 mg/kg) produced cardiorespiratory arrest, due to its hemolytic activity, associated with hyperkalemia [8,42]. A possible *in vivo* effect of APS12-2 on skeletal muscle contraction could therefore not be observed *in vivo*, since APS 12-2 produces cardiac arrest and the death of experimental animals due to its hemolytic activity and hyperkalemia at much lower doses (11.5 mg/kg), as expected for a skeletal muscle contraction block (*i.e.*, as shown by the calculated non-hemolytic median inhibitory dose (ID_50_) of 37.25 mg/kg for APS3-induced skeletal muscle contraction *in vivo*). In addition, the relative permeability of skeletal muscle capillary pores to substances with an m.w. of approximately 17 kDa (close to that of APS12-2) is ten times lower (at 0.03) than in APS12-2, so that the diffusion of APS12-2 is expected to be much slower.

### 4.2. In Vitro Physiological and Pharmacological Effects of APS12-2 and APS3

Based on the structure and anti-AChE activities of APS12-2 and APS3 (both are quaternary ammonium compounds), effects on neuro-muscular transmission were expected. sAPS are structurally related to quaternary ammonium compounds, like physostigmine, *bis*(7)-tacrine and BW284c51, some of which have dual effects and, in a concentration-dependent manner, inhibit either AChE or nicotinic acetylcholine receptors (nAChR) [50,51,52,53]. The effects on neuro-muscular transmission were revealed by experiments with both analogues on neuromuscular preparation [42,49]. APS12-2 and APS3 block nerve-evoked isometric muscle contraction in a concentration-dependent manner [42,49]. To determine their molecular mechanisms of action, the microelectrode technique on mouse hemidiaphragm preparations was applied in order to study the effects of APS12-2 and APS3 on skeletal muscle fiber resting membrane potential (RP), miniature endplate potential (MEPP) and evoked endplate potential (EPP). The direct influence of sAPS analogues on nAChRs expressed on *Xenopus* oocytes was also studied. Both analogues decreased the amplitude of EPPs and MEPPs in a concentration-dependent manner, indicating that their action may be on nAChRs [42,49]. To confirm the possibility of the direct effects of APS12-2 and APS3 on muscle-type nAChRs at the neuromuscular junction, experiments were performed on *Xenopus laevis* oocytes into which Torpedo (α2β1γδ) muscle-type nAChRs had been incorporated. It was proven that APS12-2 (IC_50_ = 0.0005 μM) and APS3 (IC_50_ = 0.19 μM) effectively block the acetylcholine-evoked current through the muscle-type nAChRs expressed in oocyte membranes, due to nAChRs inhibition [42,49].

In order to study the effects of APS12-2 and APS3, to better establish the mechanisms of their cardiovascular effects and to provide more data on mechanism specificity, experiments were performed on isolated porcine coronary vessels. In contrast to APS3, which displayed no effect, APS12-2 induced the contraction of coronary ring preparations in a concentration-dependent manner (at 1.36 to 13.60 μM). Lanthanum chloride, a non-selective cation channel blocker [54,55], and verapamil, a selective antagonist of l-type voltage-dependent calcium channels [56], completely abolished the contraction of coronary rings induced by APS12-2. This indicates that, due to increased Ca^2+^ influx through the voltage-gated Ca^2+^ channels, APS12-2 induces vascular smooth muscle contraction in a concentration-dependent manner. These results show, for the first time, that APS12-2 induces a concentration-dependent contraction of coronary ring preparations. Coronary vasoconstriction, as well as hyperkalemia, may contribute to the cardiotoxic effects of APS12-2. It is notable that the maximal final concentration of APS12-2 (13.60 μM) that produces a significant increase in coronary ring tension *in vitro* is comparable to the maximal concentration of APS12-2 in blood plasma *in vivo* following the administration of one LD_50_, which produced arrhythmia and cardiorespiratory arrest [57].

The effects of APS12-2 and APS3 on the *in vitro* measured parameters are summarized in Table 4.

**Table 4:** Physiological and pharmacological effects of APS12-2 and APS3 *in vitro*.

Measured Parameters		APS12-2 *		APS3 **	
		Effect	IC_50_	Effect	IC_50_
Skeletal muscle contraction	Nerve-evoked stimulation	Inhibition	0.74 μM	Inhibition	20.3 μM
	Direct stimulation	No effect up to 2.72 μM	N/A	No effect up to 20.55 μM	N/A
Pharmacological effect	atropine	No effect up to 80 μM	N/A	No effect up to 80 μM	N/A
	neostigmine	No effect up to 1 μM	N/A	No effect up to 1 μM	N/A
	3,4-DAP	Stops muscle contraction blockade (300 μM)	N/A	Stops muscle contraction blockade (300 μM)	N/A
Effect on	RP	No effect up to 3.40 μM	N/A	No effect up to 68.49 μM	N/A
	MEPP	Amplitude decrease, MEPP disappear above 0.68 μM	N/A	Amplitude decrease, MEPP disappear above 6.85 μM	N/A
	EPP	Amplitude decrease	0.36 μM	Amplitude decrease	7.28 μM
nAChRs inhibition		Inhibition	0.0005 μM	Inhibition	0.19 μM
Effect on coronary rings ***		Contraction (4.1–13.6 μM)	N/A	No effect up to 137 μM	N/A

N/A, Not applicable; 3,4-DAP, 3,4-diaminopyridine; RP, resting membrane potential; MEPP, miniature endplate potential; EPP, endplate potential; nAChRs, nicotinic acetylcholine receptors; * [49]; ** [42]; *** [57].

Their hypotensive action, hemolytic activity (of some compounds) and cytotoxic activity may limit the use of these substances as anti-tumor therapeutics and anti-cholinergic drugs. These effects are expressed *in vitro* at very low concentrations of APS12-2. However, relatively high doses of the tested compounds have to be used to see these effects *in vivo*, which makes these compounds suitable for preclinical testing. In conclusion, the *in vivo* toxicity of APS3 is probably the result of the reversible antagonistic action of the compound on nAChRs on motor endplates, as shown in *in vivo* and *in vitro* experiments. On the other hand, the toxicity of hemolytically active APS12-2 is probably related to the high blood potassium levels and cardiac arrest or to its direct functional effects (mechanical dysfunction) of APS12-2 on the heart conduction system. This remains to be proven. The coronary vasoconstriction produced by APS12-2 constitutes an important mechanism that can contribute to the cardiotoxicity of APS12-2. In general, the toxicity of tested sAPS is relatively low, when compared to that of natural poly-APS. The sAPS, in particular those that are non-hemolytic, are of interest for preclinical testing as novel lung tumor chemotherapeutics.

## 5. Conclusion

Synthetic APS exert a wide range of interesting biological activities that can vary according to their structure. It was shown that some of them inhibit the growth of lung cancer cells lines, either by inducing apoptosis or by inhibiting cell division. The putative underlying mechanism might be the block of the cholinergic system, which is physiologically important for lung cancer cells homeostasis. Therefore, sAPS could be suitable especially as a new class of chemotherapeutic drugs for treating non-small cell lung cancer. In recent studies, it was shown that sAPS have low toxicity that encourages their further investigation and testing as anticancer drugs. The antitumor effects of one of sAPS (APS8) are currently being preclinically evaluated on a lung carcinoma rodent model and show some very encouraging results. Synthetic APS could also find their use as agents allowing the stable transfection of cells, which could lead to their potential applications in medicine and cell biology. Finally, due to their ability to inhibit the settlement of marine organisms to submerged surfaces, they could be potentially used as active components of antifouling paints.
